# Ticks, Hair Loss, and Non-Clinging Babies: A Novel Tick-Based Hypothesis for the Evolutionary Divergence of Humans and Chimpanzees

**DOI:** 10.3390/life11050435

**Published:** 2021-05-12

**Authors:** Jeffrey G. Brown

**Affiliations:** Independent Researcher, Saddle Brook, NJ 07663, USA; Brown.jeffreyg@gmail.com

**Keywords:** forest fragmentation, parasites, last common ancestor, bipedalism, knuckle-walking, social grooming

## Abstract

Human straight-legged bipedalism represents one of the earliest events in the evolutionary split between humans (*Homo* spp.) and chimpanzees (*Pan* spp.), although its selective basis is a mystery. A carrying-related hypothesis has recently been proposed in which hair loss within the hominin lineage resulted in the inability of babies to cling to their mothers, requiring mothers to walk upright to carry their babies. However, a question remains for this model: what drove the hair loss that resulted in upright walking? Observers since Darwin have suggested that hair loss in humans may represent an evolutionary strategy for defence against ticks. The aim of this review is to propose and evaluate a novel tick-based evolutionary hypothesis wherein forest fragmentation in hominin paleoenvironments created conditions that were favourable for tick proliferation, selecting for hair loss in hominins and grooming behaviour in chimpanzees as divergent anti-tick strategies. It is argued that these divergent anti-tick strategies resulted in different methods for carrying babies, driving the locomotor divergence of humans and chimpanzees.

## 1. Introduction

The evolutionary split between humans (*Homo* spp.) and chimpanzees (*Pan* spp.) is generally agreed to have occurred in the equatorial rainforest of East Africa approximately 5–8 million years ago (Ma) [1,2,3,4]. According to one hypothesis (the “Hylobatian Model” [5]), the last common ancestor (LCA) of humans and chimpanzees was a small- to medium-sized gibbon-like ape [5,6,7,8] that lived exclusively in the upper reaches of closed forest canopies, where it fed primarily on fresh fruit [9]. Similar to modern gibbons (*Hylobates* spp.), the LCA travelled through the canopy using a combination of below-branch forelimb swinging (i.e., brachiation) and above-branch bipedal walking [5,10]. A global climatic trend of drying and cooling resulted in the fragmentation of equatorial rainforests and the emergence of a mosaic landscape comprising woodlands and grasslands [11,12]. Unable to traverse the gaps in the canopy, the LCA was forced to descend to the ground to travel between forest fragments [13,14]. Consequently, two separate lineages diverged: the chimpanzee lineage, which continued to feed at the canopy level and adopted quadrupedal knuckle-walking as its preferred mode of terrestrial locomotion, and the human lineage, which walked upright on the ground on extended lower limbs and eventually moved into more open environments.

Many hypotheses have been proposed to explain the selective basis for human bipedalism [15], including those that suggest that bipedalism evolved as a thermoregulatory response to living in more open environments [16,17], an energy-efficient means of moving between forest patches (based on comparative human–chimpanzee locomotor energetics) [18], a threat display [19], a feeding posture [20], a fighting posture [21], or an adaptation to wading in water [15]. Although most of these hypotheses provide valuable insights into the potential benefits of upright walking, they have often failed to draw a strong connection with the ecological context of forest fragmentation or explain why similar changes did not occur in chimpanzees [19].

One popular hypothesis proposes that bipedalism evolved to free the arms for carrying food or offspring [22,23,24]. Recognizing that non-human primate babies cling to their mothers by tightly grasping their mother’s hair, several researchers have suggested that hair loss in the hominin lineage could have resulted in a bipedal posture if mothers were required to use their forelimbs to carry non-clinging babies [25,26,27,28]. The safe carriage of non-human primate babies is at least in part related to the strength and density of the mother’s hair [28], and primate mothers manually support young infants that cannot yet safely cling [29,30,31]. Nonetheless, given the functional benefits of primate hair and its preservation in the chimpanzee lineage [32], the selective basis for hominin hair loss is not adequately explained by this model.

The “ectoparasite hypothesis” proposes that hair loss evolved in the hominin lineage to improve the detection of ectoparasites such as ixodid ticks (Acari: Ixodidae; hereinafter referred to as “ticks”) [32,33,34,35,36]. Because ticks are ground-dwelling parasites that commonly reside at the edges of forests [37], the LCA would have avoided contact with ticks, provided that it remained in an arboreal environment [38]. However, in descending to the ground to move from one forest patch to the next [14], the LCA would have likely encountered ticks as it necessarily passed through forest edges.

In recent years, there has been increased interest in host-parasite interactions and the potential role of parasitic diseases in influencing the evolution of their hosts [39,40,41]. Previous data have suggested that parasitic diseases may drive host diversification when non-interbreeding host populations evolve different mechanisms for disease resistance [42,43,44]. For example, human exposure to the malaria parasite *Plasmodium falciparum* approximately 10,000 years ago led to different genetically based mechanisms of malaria resistance in geographically isolated locations, including haemoglobin S in sub-Saharan Africa, beta-thalassemia in the Mediterranean and ovalocytosis in Southeast Asia [43,45,46,47]. In a similar manner, exposure of the LCA to tick-borne diseases in geographically isolated host populations may have driven selection for hair loss in humans [32,36] and grooming behaviour in chimpanzees [35,48,49] as evolutionarily divergent anti-tick strategies.

Although ancient observers recognized the harmful nature of parasitic ticks [50], and Darwin discussed their potential role in selection for hominin hair loss [51], tick ecology has rarely been considered in modern studies of human evolution [32,35,36]. The aim of this review is to propose and evaluate a novel tick-based evolutionary hypothesis wherein forest fragmentation in East Africa at approximately 5–8 Ma exposed a canopy-dwelling LCA to ground-dwelling ticks due to (1) the LCA descending to the ground to travel between forest fragments and (2) environmental changes at the edges of forest fragments favouring tick proliferation. In the chimpanzee lineage, tick exposure favoured increased social grooming with hair preservation; thus, babies continued to cling to their mothers, and the mothers’ forelimbs remained free for climbing from the ground into the canopy. Eventually, this lineage adopted relatively inefficient terrestrial quadrupedal knuckle-walking due to selection driven by quadrupedal locomotion for climbing branchless vertical supports (e.g., tall, branchless tree trunks). In the human lineage, tick exposure favoured a conserved low LCA social grooming time combined with hair loss over most of the body; therefore, babies were unable to cling to their mothers, and mothers were required to use their forelimbs to carry their babies. The mothers’ decreased ability to climb vertical supports and increased ground dwelling may have favoured straight-legged terrestrial bipedalism as an energy-efficient method for carrying loads on solid ground. This new, synthetic, tick-based hypothesis prompts re-evaluation of the scientific literature on topics pertaining to human evolution and ticks [52] and was pursued to provide a novel perspective that could contribute to the field.

To evaluate the tick-based hypothesis, this manuscript provides an in-depth discussion of the ecology of ticks, their relationships with fragmented habitats and their roles as disease vectors, as well as evidence suggesting that early hominins were exposed to ticks in fragmented paleoenvironments. In addition, the manuscript discusses the role of hair loss in tick visualization, the timing of hominin hair loss, and chimpanzee grooming as an anti-tick strategy. The paper also provides a plausible mechanism linking the carrying of babies to the divergent locomotor habits of humans and chimpanzees. Finally, limitations of the proposed tick-based hypothesis and directions for future hypothesis testing are discussed.

## 2. Forest Fragmentation and Ticks

### 2.1. Forest Fragmentation in Hominin Paleoenvironments

Forest fragmentation is the process by which a large continuous area of forest becomes fragmented into smaller patches [53]. By definition, these patches are separated by a non-forest “matrix” [54]. During hominin evolution approximately 5–8 Ma, forest fragmentation occurred in East Africa due to climatic drying and cooling, with the matrix consisting of woodlands, bushlands, and grasslands [55,56,57].

Palaeontologists have long recognized that forest fragmentation in hominin paleoenvironments coincided with early human evolution; however, the precise role of such fragmentation is not well understood [58,59,60,61]. In general, primates inhabit tropical and subtropical forests [62], and their morphological and behavioural adaptations are related to life in the trees [63]. As suggested by Darwin, the hominin lineage may have evolved when ancestral hominins began to spend less time in the trees and more time on the ground following a change in their diet and/or surrounding conditions [51]. The most recent paleoenvironmental reconstructions show that the earliest hominin ancestors, including *Orrorin tugenensis*, *Ardipithecus kadabba*, and *Sahelanthropus tchadensis*, lived in well-wooded, albeit discontinuous, forest environments [64,65,66,67,68].

Forest fragmentation results in at least two changes to the landscape that may have affected hominin evolution:

(1) Fragmentation creates barriers for forest animals, requiring them to cross through the surrounding matrix to travel from one forest patch to the next [53,69]. The matrix acts as a filter that favours the survival of specific populations [53,70,71,72].
(2) Fragmentation markedly increases the length of the forest edge (i.e., forest perimeter) [73,74,75], creating an “edge environment” that supports the influx of non-forest edge-adapted plant and animal species [69,76,77].

Arboreal primates are highly susceptible to forest fragmentation due to the patchy distribution of fruit-bearing trees and their need to access large forest areas [54]. For example, although gibbons are arboreal and normally remain within the canopy [40], they may descend to the forest floor when crossing gaps in fragmented habitats [78,79,80,81]. On the ground, primates are exposed to predators and diseases that they would not normally encounter in an arboreal environment [49,79,82,83].

“Edge effects” are the changes that occur at forest edges due to the abrupt transition between the forest and another ecosystem. These effects include changes in temperature, humidity, and light intensity [75]. The exposure of the ground to direct sunlight enables the growth of dense foliage along the forest margin [84,85]. This foliage provides food and cover for edge-adapted animals such as small mammals and ungulates [74,86,87], which may also benefit from decreased predation and competition in small forest fragments [88,89,90]. In turn, these animals create a stable host population for parasitic organisms such as ticks [91]. Ticks are abundant along large animal pathways, where they wait to attach to passing hosts [92]. Therefore, arboreal ancestors crossing gaps between patches would likely encounter ticks at the forest edge, especially if they travelled along animal paths [93].

### 2.2. Importance of Ticks

Ticks are free-living, 2- to 20-mm-long, flightless, blood-feeding parasites that are widely distributed throughout the world, living primarily in moist, humid environments [94,95]. They parasitize a wide variety of terrestrial vertebrates, including mammals, reptiles, and ground-dwelling birds [37,95]. The tick life cycle consists of three stages: larva, nymph, and adult [96]. At each stage, the tick feeds on a vertebrate host before detaching and moving back into the open environment [95]. Some ticks feed on one or two hosts during their life cycle, although three-host ticks are the most common [97]. In the three-host cycle, the tick feeds on three separate hosts to complete its life cycle [96]. In the adult stage, a male and female mate on the host. The adult female feeds to engorgement and then drops to the ground to lay eggs, typically over a thousand, which then hatch into larvae [95,96]. Ticks consume one blood meal per life stage and may enter a period of quiescence to optimize the timing of their feeding activity, enabling many tick species to survive for longer than a year before their next blood meal [98].

After digesting the blood meal and advancing to the next stage of its life cycle, the tick “quests” for its next host, in which it ascends a blade of grass or low vegetation and waits for an animal to pass [95]. When the tick senses a host via vibrations, body heat, exhaled carbon dioxide, or other factors [94,99], it extends its forelimbs in preparation to cling to the passing host [96,97]. Once on the host, the tick identifies a location from which to feed, embeds its mouthparts in the skin of the host, and then feeds for a few days to over a week (depending on the stage of the tick) before detaching and re-entering the environment [94].

Ticks show varying degrees of host specificity and may seek different species during each part of their life cycle [98,99]. For example, *Ixodes scapularis*, the principal vector of Lyme disease in North America, feeds on white-footed mice (*Peromyscus leucopus*) in its immature stages and on white-tailed deer (*Odocoileus virginianus*) as an adult [88]. In general, ticks use greater heights on vegetation to access larger hosts [91,98], ranging from ground level to approximately 1 m high [97,100,101]. Ticks are susceptible to dehydration and travel only a short distance from the more humid air near the ground, where they periodically return to absorb ambient water [95].

Ticks can transmit a wide range of infectious agents, including bacteria, viruses, and protozoa [95], which they acquire when feeding on an infected host [102]. Some pathogens can also be transmitted transovarially from mother to offspring, thereby enabling multiplication of the disease vector [102]. Although the number of microbes required to establish an infection varies [103], for most transmitted diseases, the bite of a single infected tick is usually sufficient to infect the host [102].

Pathogen transmission typically requires 20–48 h [104,105,106,107], although some agents may be transmitted more rapidly [103,108]. This delay in pathogen transmission combined with the time required by ticks to explore the host and locate a suitable feeding location [108] creates a window of opportunity for the early detection and removal of ticks [107], such as by grooming [109].

The ecology of ticks and epidemiology of tick-borne diseases are closely linked to the behaviour and characteristics of their small and large mammalian hosts [37]. Small mammals (defined as mammals weighing <3 kg as adults [110]), particularly rodents, play an important role in supporting the immature stages of numerous tick species and act as pathogen reservoirs [37]. Large mammals (weighing >3 kg as adults [110]) represent a stable blood-meal source for the adult stage of numerous tick species, serve as a rendezvous site for tick mating, and disperse ticks over long distances [37,111].

### 2.3. Ticks and Forest Fragmentation

The distribution of ticks in nature is patchy and depends on the climate, vegetation, and availability of suitable hosts [99,112,113]. Ticks cannot tolerate the high heat and low humidity of open grasslands, and they are most commonly located at the edges of forests, where dense foliage protects the ticks from sunlight and provides food for their terrestrial hosts [94,112,114]. Ticks can travel only a few metres horizontally, and they locate their next host near to where they detached from their previous host [37,97,112]. Hence, the forest edge provides ticks with a stable supply of blood-meal sources and represents a habitat to which ticks are physiologically adapted [76,97,112].

Increasing evidence supports a relationship between forest fragmentation, mammalian host populations, and the epidemiology of tick-borne diseases [74,88,115,116,117,118]. For example, studies on Lyme disease in North America have demonstrated that decreasing forest patch size (i.e., increasing fragmentation) is positively correlated with the density of *I. scapularis* nymphs and the percentage of nymphs infected with the Lyme disease bacterial agent, *Borrelia burgdorferi* [88,119]. The prevalence of *B. burgdorferi* appears to be linked to the population size of the principal disease reservoir, the white-footed mouse, which proliferates in small forest patches [88,120,121,122]. Uninfected larval ticks become infected while feeding on infected mice [88]. White-tailed deer, the principal hosts for adult ticks, are abundant in fragmented habitats and are key determinants of landscape tick density [74,111,119,123]. Humans acquire the Lyme disease agent when they enter forest fragments and become the host of an infected tick [88,119,124]. Epidemiological studies show that forest fragmentation worldwide is associated with an increased incidence of tick-borne diseases, including Lyme disease (North America, Europe, and northern Asia), tick-borne encephalitis (Europe and northern Asia), Rocky Mountain/Brazilian spotted fever (North, Central, and South America), Kyasanur Forest disease (southern India), and Crimean–Congo haemorrhagic fever (Eastern Europe, Africa, Central Asia, and the Middle East) [116,117,125].

### 2.4. Hominin Exposure to Ticks in Fragmented Paleoenvironments

Comparisons of animals found in the fossil record with similar animals found today and studies on the ecology of these extant species enable the reconstruction of hominin paleoenvironments (i.e., taxonomic and ecological “uniformitarianism”) [40,126]. Forest fragmentation in the modern era has been associated with ticks and tick-borne diseases [88], and it is reasonable to hypothesize that a similar relationship existed in the past. The herding of large domestic animals in the modern era has provided an increased opportunity for tick proliferation [37,127,128]. In a similar manner, the expansion of the East African grasslands during the Tertiary (66–2.6 Ma) along with the expansion and diversification of several large mammalian lineages likely provided ticks with increased opportunities for expansion and evolutionary radiation [37].

The finding of spirochete-like cells in a 15 million-year-old fossilized *Amblyomma* tick in the Dominican Republic supports the ancient ancestry of ticks and their ability to transmit pathological agents [129]. There are currently no reports of ticks in the African fossil record [130,131]; however, modern Africa has an abundance of ticks, which are an important cause of human diseases and result in large economic losses to the livestock industry [127,132,133]. Of the 705 extant tick species found worldwide, 206 (29.2%) are endemic to the Afrotropical Region [113,134]. Important disease-bearing Afrotropical ticks include *Amblyomma variegatum* (“tropical bont tick”) and *Rhipicephalus appendiculatus* (“African brown ear tick”) [135]. *A. variegatum* is the principal vector of African tick bite fever and related human rickettsial infections, and *R. appendiculatus* is the principal vector for East Coast fever, or theileriosis, a protozoan livestock infection [105,136].

Fossil assemblages from early hominin sites feature large numbers of small and large mammals [137]. For example, late Miocene deposits of *Ardipithecus kadabba* from the Adu-Asa Formation, Ethiopia (5.77–5.4 Ma), are part of an assemblage that includes root rats (*Tachyoryctes* spp.), cane rats (*Thryonomys* spp.), and reduncine bovids [68,137]. Root rats and cane rats are extant rodent species that are broadly distributed throughout sub-Saharan Africa [138]. The greater cane rat (*Thryonomys swinderianus*) is parasitized by numerous tick species and is the preferred host for all stages of *Rhipicephalus simpsoni*, a three-host tick [113]. Reduncine bovids (e.g., reedbucks and waterbucks) have been documented as hosts for several tick species, including the disease-bearing ticks *A. variegatum* and *R. appendiculatus* [139]. Fossil assemblages from Toros Menalla, Chad (*Sahelanthropus tchadensis*), and Tugen Hills, Kenya (*Orrorin tugenensis*), also feature large numbers of bovids [137]. The finding of early hominin remains in association with endemically parasitized mammalian species supports the proposed hypothesis that early hominins were exposed to ticks.

It is important to note that ticks and their infectious agents exist in ecological balance with their natural terrestrial hosts (due to long-standing coevolutionary relationships) [37,140,141,142] and that terrestrial hosts can act as pathogen reservoirs without becoming diseased [140,143]. In contrast, an arboreal LCA that descended to the ground and encountered ticks would have been exposed to tick-borne disease as part of a novel disease cycle (i.e., a zoonosis) for which it would lack natural resistance [49]. Novel pathogens that enter naïve populations often result in mass mortality, potentially driving selection for sub-populations with increased disease resistance [144,145]. Studies show that cattle breeds native to Africa have increased levels of tick resistance compared with breeds imported from Europe, a finding likely due to long-term natural selection driven by ticks (during the early 1900s, European cattle in Southern Africa suffered >90% mortality following outbreaks of tick-borne diseases [146]) [147,148]. In a similar manner, LCA exposure to ticks in fragmented paleoenvironments may have driven selection for LCA sub-populations with increased tick resistance. Because natural selection can potentially result in more than one successful strategy [46,149], hair loss in hominins and social grooming in chimpanzees may therefore represent two sub-populations derived from their LCA with divergent adaptations for defence against ticks.

## 3. Evolution of Alternative Anti-Tick Strategies

### 3.1. Hominin Hair Loss

Hair is an epidermal appendage that has several important functions, including insulating the body from heat loss and protecting the skin from ultraviolet radiation [150]. Given these important benefits, humans are unique among primates due to their relative lack of body hair [40,151]. Humans and chimpanzees have a similar number of hair follicles [152]; however, while chimpanzee body hairs are long, thick and pigmented (i.e., terminal hairs), human body hairs are short, thin and transparent (i.e., vellus hairs) [153], providing the human body with little insulation or protection [151].

Several hypotheses have been proposed to explain the adaptive basis of hominin hair loss, such as hair loss being an adaptation allowing the dissipation of heat in open environments (in conjunction with sweating) [16], a sexually selected trait [51], an adaptation to aquatic environments [154], or a protective measure against ectoparasites, primarily lice and ticks (i.e., the “ectoparasite hypothesis”) [32,33,35,36]. Because non-human primate lice are rarely associated with infectious diseases and in small numbers do not cause serious harm [155], the more likely factor for the “ectoparasite hypothesis” is exposure to disease-bearing ticks. Ticks can adhere directly to skin and do not require hair to cling [156]; rather, hair loss may have contributed to tick control by allowing ticks to be more easily seen and removed [34].

The role of hair loss in tick visualization is supported by the observation that dense hair is visually opaque and shields the skin from view [157,158]. For example, skin pathologies or irregularities can be concealed under hair [159,160], and hair removal improves skin visualization [161]. The unfed ticks of most tick species range in size from 2 to 7 mm [162], and tick bites are generally painless [163]; thus, feeding ticks could likely go unnoticed on skin that is covered with hair. The role of hair in obscuring ticks is supported by studies in cattle in which the use of infrared thermography to quantify ticks was found to be limited by overlying hair [164,165]. In addition, children suffering from undiagnosed cases of tick paralysis (caused by neurotoxin-releasing ticks) have been found after several days to have an engorged tick that is attached to the scalp and concealed by the hair [166]. Tick visualization following hair loss may have been enhanced by pale skin [prior to the evolution of increased melanin in more open environments [167]] and an absence of clothing [168]. Moreover, tactile perception of tick movement may have been enhanced by short, fine hairs [169].

#### Evolutionary Timing of Hominin Hair Loss

Accepting that hair loss improves tick visualization and allows more efficient tick removal, support for the tick-based hypothesis requires consideration of the evolutionary timing of hominin hair loss. According to the tick-based hypothesis, hominin hair loss should coincide with forest fragmentation ~5–8 Ma, the most likely paleoenvironmental context for hominin exposure to ticks.

Currently, indirect evidence from a variety of sources provides only broad, general estimates for the timing of hair loss [151,170]. For example, following the loss of hair, skin pigmentation likely increased to protect the skin against ultraviolet radiation [167]. Silent mutations in African versions of the human melanocortin 1 receptor (*MC1R*) gene, which plays a key role in regulating skin pigmentation, place hair loss as occurring prior to 1.2 Ma [170]. Alternatively, studies of hominin thermoregulation suggest that hair loss must have occurred prior to or at the time of living in more open environments (~2.0 Ma) to enable heat loss by sweating [16]. Finally, research indicates that hair loss must have preceded hominin encephalization (~2.2–2.4 Ma) based on the dietary restriction of several amino acids that are required for both hair and brain development [171]. These time ranges are not helpful in determining the date of hominin hair loss, however, since a date prior to ~2.4 Ma is still consistent with either an early (~5–8 Ma) or late (~2.4 Ma) date.

Alternatively, studies on the origin and distribution of lice, the life cycles of which are linked to a single host species and can be used to reconstruct the host’s evolutionary history, offer compelling evidence that human body hair was lost between 5–7 Ma and 3–4 Ma. Taxonomists have long recognized that humans serve as hosts to two different genera of lice (*Pediculus* and *Pthirus*) [172], with the human head louse (*Pediculus humanus capitis*) related to the chimpanzee body louse (*Pediculus schaeffi*) and the human pubic louse (*Pthirus pubis*) related to the gorilla body louse (*Pthirus gorillae*), although no explanation was offered for this interesting observation [151] [the human clothing louse, *Pediculus humanus humanus*, diverged from *P. humanus capitis* much more recently (between 170,000 to 83,000 years ago) and is not part of the story [168]]. Recently, Reed*, et al.* [173] recognized that divergence dates for the different species of lice could be used to date the evolution of hominin hair loss based on the presumption that the LCA body louse became confined to the hominin head due to hair loss over the rest of the body and that the subsequent evolution of a visual patch of pubic hair for sexual signalling created a novel ecological niche that allowed the gorilla body louse to move to hominins [151]. A divergence date of 5–7 Ma for the human head louse and chimpanzee body louse and a divergence date of 3–4 Ma for the human pubic louse and gorilla body louse support the conclusion that hominin body hair was lost between 5–7 Ma and 3–4 Ma [151]. This early time frame is consistent with the view that hair loss occurred when hominins still occupied fragmented forests (also the most likely location for contact with gorillas [151]) [28] and supports the tick-based hypothesis.

An early date for hominin hair loss is also supported by interspecific comparative studies of hair density, in which chimpanzees were found to have a particularly low density of terminal hairs compared with other primates after adjusting for body size [174]. The presence of hair loss in both the human and chimpanzee lineages, albeit to different degrees, suggests that the evolutionary pressures resulting in hominin hair loss may have initially acted on both lineages or even the LCA [152,174] (perhaps due to a phase of terrestrial exposure just prior to the split), thus supporting the view that hominin hair loss occurred close to the time of the human–chimpanzee divergence at 5–8 Ma [174].

In opposition to the early timing of hominin hair loss as proposed by the tick-based hypothesis, recent thermoregulatory considerations suggest that hair loss would not have been possible when early hominins lived in high-altitude fragmented forests (above ~1000 m above sea level) due to low night-time temperatures (5–10 °C); therefore, hair loss must have occurred at a later time (~2.0 Ma), when hominins moved into more open environments closer to sea level [16]. This argument is compelling, and it is reasonable to ask how early hominins tolerated low night-time temperatures without the thermoregulatory benefits of hair. Although there is no way to be certain, it should be noted that much of the fossil and archaeological evidence of early hominins in East Africa has been found at sites located directly adjacent to large paleolakes [175], supporting the view that at least some hominins inhabited these areas. Lakes can affect the regional climate by absorbing daytime solar radiation and releasing this energy as heat at night [176], which may have helped to offset low night-time temperatures in high-altitude lakeside forest environments. Modern East African climate data for multiple sites surrounding Lake Victoria (range 1130–1526 m above sea level) collected between 1980 and 2016 show that annual lowest night-time temperatures range from 12 to 16 °C [177] (Supplementary File S1), which is considerably warmer than 5–10 °C, as previously reported. Pliocene temperatures may have also been warmer by 2–3 °C [16]. In addition, it is possible that early hominins developed behavioural adaptations such as building warmer nests [178], huddling together for warmth at night [179], or nesting in more closed forest areas, which are typically warmer than open areas during the night [180]. Finally, compared with other primates, human babies appear to have unique adaptations for preserving or producing heat, such as a large birth size (resulting in a decreased surface to body mass ratio) [181,182], thickened subcutaneous insulating white fat [183], and abundant deposits of heat-producing brown fat [184]. Although the evolutionary history of these thermoregulatory adaptations has not been well characterized (data on brown fat in apes are currently lacking [185]), studies suggest that Australopithecine mothers (pre-encephalization [186,187]) already birthed large babies [181], supporting the view that at least some of these features may have evolved to protect hominin babies from hypothermia due to the loss of hair [182]. Hence, although hominin hair loss would represent a thermoregulatory disadvantage for tolerating low night-time temperatures in high-altitude forests, given other considerations, the possibility that hair loss evolved early in these cooler environments cannot be excluded.

Finally, it is worth asking why hairlessness did not evolve in other taxa (e.g., terrestrial ungulates) despite prolonged exposure to ticks [51]. This question might be addressed by considering the roles of determinism and contingency in evolution; although there are many examples of convergent evolution in which similar outcomes result from similar selection pressures (i.e., determinism), there are also examples in which the evolutionary outcomes are different, perhaps due to specific circumstances or events (i.e., contingency) [188]. For example, given that ungulates have evolved immunologic [189] and oral grooming [190] defences against ticks, it is possible that hair loss (given other disadvantages) may not have provided sufficient benefit to promote this evolutionary pathway. By contrast, hominin hair loss would have evolved in a tick-naïve arboreal primate possessing a high level of manual dexterity [191] and visual acuity [192]. Accepting low social grooming times in early hominins (conserved from the LCA), hair loss in the hominin lineage may have provided a selective advantage by revealing otherwise concealed ticks [34].

### 3.2. Chimpanzee Grooming

Social grooming (i.e., allogrooming) is ubiquitous among non-human primates and serves several important social functions, including group cohesion, tension reduction, mother–infant bonding and acting as a courtship ritual [31,193,194,195]. However, there is also evidence to support the view that social grooming serves a primarily hygienic function, particularly in the defence against blood-feeding ectoparasites such as lice and ticks [34,48,49]. For example, while self-grooming targets areas accessible to the individual, social grooming primarily targets areas inaccessible to the individual, such as the head, neck, back, and rump [194,196,197,198].

Lice and ticks have distinct ecologies and life cycles that have important implications for primate social grooming. While lice (Insecta, Phthiraptera) are species specific, spend all of their time on the host, and are uncommonly associated with infectious agents [199], ticks (Arachnida, Ixodidae) move between different host species, spend most of their time off the host, and are able to transmit a broad range of infectious agents [200]. Nearly all primate species serve as natural hosts for lice [201] (with the exception of the orangutan (*Pongo* spp.), the solitary lifestyle of which may not permit louse survival [173,202]); however, only primates that spend time on the ground are exposed to ground-dwelling ticks [38,203,204]. For example, Kyasanur Forest Disease in southern India is transmitted to langurs (*Semnopithecus* spp.) and bonnet macaques (*Macaca radiata*) when primates foraging on the ground are exposed to infected larval ticks [205,206]). Because ticks are generally harmful to their hosts and can serve as a source of infectious agents [207], primates with terrestrial exposure to ticks would be expected to have an increased duration and intensity of social grooming.

Previous findings have shown that primates naturally acquire ticks in terrestrial environments [109,204] and that tick burdens increase with time spent on the ground [38]. For example, mouse lemurs (*Microcebus griseorufus*) have been found to harbour ticks during only the dry season (May–October), the period of time when they descend to the ground [38]. Compared to females, male mouse lemurs spend more time on the ground foraging and travelling and suffer higher rates of tick infestation [38]. Similarly, baboons (*Papio* spp.) are a primarily terrestrial species [208] that also suffer from large tick infestations. In one study, 62% (40/65) of baboons were found to harbour one or more ticks, with an average tick burden of 39 ticks/individual [109].

Published data and observations support a relationship between tick infestation and primate social grooming. In baboons, tick burdens strongly correlate with the degree of social grooming, with younger, female, and higher-ranking adult individuals receiving the most grooming and harbouring the lowest number of ticks [109]. Solitary baboons and langurs (*Semnopithecus* spp.) have been observed to be heavily infested with ticks compared with group-living conspecifics [209], with one solitary male baboon harbouring over 200 ticks [210].

Chimpanzees are highly motivated social groomers [197] that spend a large portion of their daily activity (i.e., their awake time) on the ground (average of 60.2% of their daily activity during the dry season (calculations based on Table III in Wrangham [211])). Ticks have been reported in chimpanzee environments [203,212], whereas chimpanzees themselves are rarely found to have ticks [213]. Indeed, chimpanzees appear to have low rates of tick infestation since grooming activity is not noticeably different during the months when environmental ticks are most prevalent [212]. Although these data could be used to argue that chimpanzee grooming is not related to ticks and serves a mostly non-hygienic (i.e., social) function [214], it is possible that tick infestation was more common in the past (when the climate was warmer and more humid [55]) and/or that modern chimpanzees have behavioural or physiological adaptions that help reduce tick exposure. It is also possible due to the efficiency of chimpanzee grooming and the low probability of obtaining a blood meal that ticks in chimpanzee environments have evolved to avoid chimpanzees.

Ticks observed on chimpanzees are soon removed by grooming [31]. The discovery of a novel species of tick that feeds within chimpanzee nostrils (where the ticks cannot be manually accessed) may represent the evolution of an anti-grooming countermeasure by ticks [213]. Ticks take time to explore their host [108], and there is a delay in transmission of approximately 1–2 days for most infectious agents [104,105,106,107]; therefore, a daily grooming session would likely be sufficient to protect chimpanzees from most tick-borne diseases [215]. Because a single bite from an infectious tick can cause severe illness or death in an otherwise healthy individual [216], and non-infected ticks in sufficiently large numbers can cause host anaemia and death or a general failure to thrive [204,217], protection from ticks would be expected to provide a strong selective benefit for social grooming [218,219].

Grooming by common chimpanzees (*Pan troglodytes*) from Gombe, Taï, and Mahale accounts for 6.2%, 9%, and 14.1% (average 9.8%) of their daily activity, respectively, and grooming by bonobos (*Pan paniscus*) from Lomako accounts for 5.7% of their daily activity. The average chimpanzee grooming time based on the two *Pan* species is 7.8% (Supplementary File S2), a value similar to that observed for terrestrial Old World monkeys (9.2%) and terrestrial lemurs (7.8%) [220]. In contrast, predominantly arboreal primates, including orangutans, New World monkeys, gibbons/siamangs, and some species of Old World monkeys and lemurs show lower grooming times accounting for 0.01%, 1.9%, 3.9% (range: 0–10%), 3.7% and 4.1% of their daily activity, respectively [220].

In humans, a study of six traditional small-scale societies showed that anti-parasite social grooming time (mostly directed at removing lice from the scalp) accounted for an average of 0.8% of their daily activity, a value significantly lower than expected when compared to typical primates [221]. Accepting that the LCA was an arboreal primate that was also a social groomer, this finding suggests that modern human social grooming time represents a decrease of the LCA’s social grooming behaviour, perhaps due to visual inspection replacing social grooming after hair loss exposed the skin. In contrast, high social grooming time in chimpanzees likely represents an increase of the LCA’s arboreal social grooming behaviour. Decreased social grooming time with hair loss in hominins and increased social grooming time with hair preservation in chimpanzees may therefore represent divergent evolutionary responses following the LCA’s terrestrial exposure to ticks. Notably, gorillas and François’ langurs (*Trachypithecus francoisi*) have low social grooming times (1.3% and 1.2%, respectively) despite living on the ground (gorillas inhabit tropical forests [222], and François’ langurs inhabit tropical limestone cliffs [223]). These data suggest that these primates may not be exposed to large numbers of ticks in their local environments or that they have evolved alternative non-grooming anti-tick strategies that are currently unknown.

#### The Social Brain Hypothesis for Primate Allogrooming

An alternative hypothesis (the “social brain hypothesis”) proposes that primate social grooming was initially hygienic but further evolved in terrestrial environments to help protect against predators, by maintaining social bonds, enhancing group cohesion, and increasing group size [214,224,225,226,227,228]. These social effects may have been facilitated by endorphin-mediated neural pathways in which primates received pleasure while being groomed [228,229,230,231]. Although this hypothesis has merit (chimpanzees actively solicit social grooming by presenting a part of their body to be groomed [31]), the motivation to receive social grooming applies to the groomee (i.e., the individual being groomed) but not necessarily the groomer (i.e., the individual performing the grooming). For example, while the chimpanzee groomee sits quietly and cooperatively [214,232], consistent with a tendency towards communal behaviour, the chimpanzee groomer typically focuses on the activity being performed [31,197,232]. Using a hand or lower lip, the groomer parts the hair in the area of interest and uses the opposite hand to probe the exposed skin [31,233]. Abnormal findings are removed with the lips or pinched between the index finger and thumb [31]. These coordinated movements of the hands, fingers, and lips [197,232] do not appear to have a social function; rather, they are more likely related to the efficient removal of ectoparasites [34].

The chimpanzee groomer’s activity is also characterized by close visualization of the area being groomed [31,197,233]. For example, the distance between the groomer’s eyes and fingers (i.e., “the grooming distance”) varies with the groomer’s age [234], with younger chimpanzees focusing at a distance of approximately 10–20 cm, and older chimpanzees (>45 years old) focusing at a distance of 40–50 cm, a finding consistent with farsightedness (presbyopia) in older individuals [234,235]. The observation that older chimpanzees adjust their posture and extend their arms to groom at longer distances (they close the gap when applying their mouth to the skin) [234,235,236,237] supports the role of vision in grooming and indicates a primarily hygienic, non-social function. Consistent with a relationship between seeing and grooming, a study performed in common marmosets (*Callithrix jacchus*) living in captivity has shown that self-grooming normally depends on having adequate levels of ambient light (200–500 lux) [238]. Notably, nocturnal primates that normally groom in the dark (e.g., lorises) do not rely on vision; rather, they grip their partner’s skin with their hands and groom using their mouths [239].

Chimpanzee groomers have been observed to surge with excitement when encountering a louse or a tick [31,197,212] by loudly smacking their lips and clacking their teeth [240,241] in a response described as a “jackpot effect” [197]. The parasite is typically removed, intensely observed, crushed, and then consumed using exaggerated movements of the jaws [197,240,242]. Indeed, the groomer usually “lip-smacks’ and “tooth-clacks” throughout the grooming activity [232,241], even in advance of finding a parasite, suggesting that part of the groomer’s pleasure may be anticipatory [243] and that chimpanzees may have inherent reward preferences for finding ectoparasites [202,244]. Given the risk associated with tick-borne diseases [245] and the likelihood that the LCA was exposed to ticks in fragmented paleoenvironments, the groomer’s careful technique in parting the hair and examining the skin supports the proposed hypothesis that chimpanzee groomers are primarily searching for ticks. Therefore, for early chimpanzees living in tick-dense environments, the groomer’s pleasure in searching for ticks may have been reinforced by the groomee’s pleasure in being groomed [34], with the additional social benefit of increased group cohesion and group size, which may have helped to protect the chimpanzees against predators [246,247,248].

## 4. The Locomotor Divergence of Humans and Chimpanzees

### 4.1. The Locomotor Repertoire of the LCA

The divergent locomotor habits of humans and chimpanzees following the proposed LCA terrestrial exposure to ticks can be best understood by first considering the locomotor habits of the LCA. As noted by Thorpe, et al. [249], the LCA is often presumed to have been a quadrupedal primate that converted to bipedalism with the origin of the human lineage. However, there is also support for the hypothesis that the LCA was already bipedal and used a bent-hip, bent-knee (BHBK) gait (i.e., with the hips and knees flexed throughout the gait cycle) as an adaptation for walking on flexible tree branches [7,81,250,251] (contra, see [252,253,254]). LCA arboreal bipedalism is hypothesized to have been either hand-assisted (e.g., in the manner of modern orangutans) [7] or hand-unassisted (e.g., in the manner of modern gibbons), with the latter hypothesis referred to as the “Hylobatian Model” [5,255]. If human bipedalism represents conservation of the LCA’s upright bipedal posture, then quadrupedal knuckle-walking in modern chimpanzees represents reversion to walking on all four extremities [7,256].

### 4.2. The Hylobatian Model for the LCA

The Hylobatian Model for the LCA was first proposed by Keith [5,10] based on his observations that when gibbons swing from or walk on branches, their bodies are oriented perpendicular to the support, in a manner similar to upright walking in humans. Field studies show that gibbons are naturally bipedal and walk upright on branches and lianas (large woody vines) for short bouts [257,258,259]. In one of these studies, bipedalism accounted for 12% of gibbon travelling locomotion [257], a mode which gibbons alternate with brachiation, their predominant means of travelling long distances (48% of their travelling locomotion in the same study) [257,260]. Gibbon bipedalism uses a BHBK gait [81], which maintains a low level center of gravity and likely represents an adaptation to minimize destabilizing vertical oscillations when walking on flexible horizontal supports [7,250,258,261,262,263]. Stability is enhanced by prehensile feet that grasp the underlying support [81] and a flexible “midtarsal break,” which enables the heel to be lifted while the metatarsals and phalanges remain on the support, allowing smooth forward travel of the foot’s center of pressure below the body’s center of mass [264,265]. A study of gibbons walking outside on a grassy substrate and inside on a walkway (n = 232 sequences) has shown that gibbons primarily walk bipedally on the ground (80% of sequences), although they sometimes use tripedal and quadrupedal gaits (8% and 12% of sequences, respectively) [81]. On the ground, gibbons maintain their use of a BHBK gait [79,80,81,258]. Gibbons have also been observed to walk bipedally when crossing gaps in the canopy [79]. Although orangutans walk bipedally in trees (in addition to other forms of locomotion) [249,266], on the ground they are generally quadrupedal [267,268]. Therefore, if the LCA moved in the trees in a manner similar to modern gibbons (although possibly at a slower speed and with greater caution [8,269]), it is reasonable to suggest that it would have also walked bipedally on the ground [10].

### 4.3. Evidence in Support of the Hylobatian Model

The genetic proximity of humans and chimpanzees seems to suggest that humans evolved from a knuckle-walking ancestor [253,270]; however, this view is called into question by anatomic studies showing that the wrists of modern humans and early hominins lack knuckle-walking features [255] (contra, see [271]). In addition, knuckle-walking features in chimpanzees and gorillas are morphologically distinct, suggesting that these features arose independently in the two lineages [272,273]. Therefore, rather than having common ancestors that walked quadrupedally on the ground, humans, chimpanzees, and gorillas may have evolved independently from bipedal arboreal ancestors. In addition to bipedal locomotion, these arboreal ancestors would have likely possessed adaptations for suspension and brachiation in trees [274]. Anatomic features of modern apes that support a brachiating ancestor include a broad, flattened ribcage (allowing greater reach when swinging from branches), posteriorly positioned scapulas, laterally directed shoulder joints and highly mobile shoulders and elbows [10,275] (contra, see [276]). Gibbons, humans, and all early hominins have relatively long, flexible lumbar spines [10,277,278,279], which could reflect the ancestral condition in which lateral flexibility allowed for greater balance when walking upright on tree branches [280]. Short stiff lower spines in gorillas and chimpanzees [277,278,279] have different morphological patterns [279] and could reflect convergent evolution related to quadrupedal knuckle-walking, perhaps as an adaptation to improve lumbar support for carrying offspring on the back [31,281]. Although evidence is fragmentary and new fossils continue to be found, the recent finding of a new Miocene ape (*Danuvius guggenmosi*, 11.62 Ma) from Bavaria, Germany, that shows adaptations for below-branch forelimb suspension and above-branch bipedal walking, supports the view that humans and chimpanzees may have evolved from a brachiating, bipedal arboreal LCA [8,269,270,274,282] (contra, see [283]).

### 4.4. Hominin Straight-Legged Bipedalism

Straight-legged bipedalism in the hominin lineage is distinct from the BHBK bipedalism of most non-human primates in that the weight of the body is continuously supported over fully extended lower limbs (alternating left and right) [284,285,286,287,288]. Because the toe-off and heel-strike components of the straight-legged gait produce large ground reaction forces [250] that naturally require a stable support [289], straight-legged bipedalism represents a form of bipedalism that is uniquely adapted for walking on the ground (i.e., not in trees). In humans, the prehensile and flexible foot of non-human primates has been converted into a rigid lever (with shortened metatarsals, a non-opposable hallux and foot bones arranged in the form of an arch), in which force exerted by the calf muscles on the calcaneus (motive force) rotates the foot forward at the metatarsophalangeal joints (fulcrum, stabilized on the ground), thereby lifting the body against gravity (resistive force) and creating a source of gravitational potential energy that is then used to propel the body over a fully extended opposite leg with each step [263,290,291,292,293,294,295]. Straight-legged bipedalism is more energy efficient than BHBK bipedalism (when walking on the ground), in part due to decreased rotational forces in the hips and knees (i.e., torques), requiring less muscular force to maintain posture [296]. This locomotor efficiency is pronounced when carrying loads [286,297,298,299]. The evolution of straight-legged bipedalism as an energy-efficient mechanism for carrying loads on the ground could help explain the paradoxical observation that humans are optimized for walking at slow speeds (0.5–1.5 m/s) [284,297,300], which would likely increase the risk of being attacked [301], although early hominins could have escaped from predators by climbing trees [302].

Growing babies become increasingly heavy and require carrying almost continuously [28,303,304]; they therefore represent substantial loads that would be expected to select for carrying efficiencies in their mothers [305]. Hair loss in hominin mothers would have made the forelimb carrying of non-clinging babies obligatory [28]. Although mothers could carry babies in their forelimbs when walking upright (accepting that the LCA was already bipedal), baby-carrying mothers with their forelimbs occupied may have been limited in their ability to climb branchless vertical supports [181,266], an activity that typically uses all four extremities [306], and thereby may have been required to feed closer to the ground. As a result, baby-carrying foraging mothers may have transitioned to small open-forest fruit trees with lower, more accessible branches [307], gradually increasing their reliance on terrestrial sources of food [308]. Male offspring with straight-legged adaptations (e.g., loss of prehensile feet) [303] would also become increasingly restricted to feeding on the ground. Therefore, in the hominin lineage, exposure of the bipedal LCA to the terrestrial environment may have favoured straight-legged terrestrial carrying efficiencies when use of the forelimbs to carry non-clinging babies limited the mothers’ ability to climb tall trees.

In contrast to the energy-efficient bipedal locomotion of humans, the evolution of relatively inefficient terrestrial knuckle-walking in chimpanzees may represent a morphological compromise due to quadrupedal adaptations for climbing branchless vertical supports (typically less than 20 cm in diameter [309]) [7,18,310,311,312] [contra, see [250]]. Whereas an arboreal LCA moving between feeding sites would have travelled within the canopy, chimpanzees descend from the canopy and travel on the ground [10,31]. Because vertical climbing is critical for chimpanzees in order to access their primary food source [309], and branchless vertical supports may be particularly dangerous to climb (e.g., rather than climbing down the tall, branchless trunk of a palm, chimpanzees may jump to a different type of tree and then descend by means of the peripheral branches [31]) [181,309,313], it is reasonable to suggest that evolutionary selection for chimpanzee locomotion has been primarily driven by the need for safe vertical climbing [310]. Chimpanzee terrestrial knuckle-walking and vertical climbing share similar postural and kinematic features, despite being directed in different planes (i.e., horizontal versus vertical): the body and the support are approximately parallel [306,314], the hips and knees are highly flexed [307,315], and the feet are positioned plantigrade against the support [31]. Propulsion is primarily generated via plantar flexion of the foot at the ankle with extension of the lower limb against the support [316,317]. While the forelimb in vertical climbing contributes by pulling with the fingers [317], the forelimb in knuckle-walking contributes by pushing with the knuckles [318]. Nonetheless, forward progression in both activities is directed parallel to the body, in contrast to the perpendicularly directed forward progression of bipedal walking [5]. Therefore, in the chimpanzee lineage, exposure of the bipedal LCA to the terrestrial environment may have resulted in quadrupedal knuckle-walking when the continued use of canopy foraging due to preserved baby clinging favoured quadrupedal locomotion for climbing branchless vertical supports.

## 5. A Tick-Based Hypothesis for the Evolutionary Divergence of Humans and Chimpanzees

**Figure 1:**
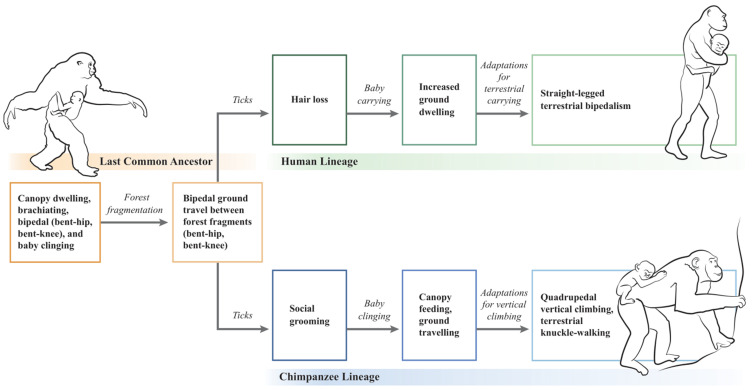
A tick-based hypothesis for the evolutionary divergence of humans and chimpanzees. Illustration by Emily M. Eng.

Following forest fragmentation and terrestrial exposure to ticks, straight-legged bipedalism in hominins and knuckle-walking in chimpanzees are hypothesized to have evolved as divergent locomotory responses to carried and clinging babies, respectively (Figure 1). In the hominin lineage, LCA bipedal walking (BHBK) evolved into straight-legged terrestrial bipedalism, whereas in the chimpanzee lineage, LCA bipedal walking (BHBK) evolved into quadrupedal vertical climbing and terrestrial knuckle-walking. According to the hypothesis, the human lineage represents conservation of the LCA’s upright bipedal posture, whereas the chimpanzee lineage represents reversion to walking on all four extremities. 

## 6. Limitations of the Tick-Based Hypothesis

The tick-based hypothesis is limited by a lack of direct evidence of ticks and tick-borne diseases in hominin paleoenvironments. Ticks and the microbes that they harbour are unlikely to fossilize [319], and the genetic identification of either would require preserved genetic material. Hence, evidence for the central component of the tick-based hypothesis (i.e., ticks) is likely to remain indirect.

A second limitation of the hypothesis is that it does not explain why selection for hair loss would act primarily on the hominin lineage, whereas selection for social grooming would act primarily on the chimpanzee lineage, following exposure to ticks. It is possible that the LCA was not a homogeneous group of individuals but rather existed in small, isolated populations with minor physical and behavioural differences. A more gregarious population may have undergone selection for social grooming, whereas a more solitary population with thinner hair may have undergone selection for hair loss. Local conditions (e.g., slightly warmer or cooler temperatures) may have also favoured one pathway over another. Nonetheless, given that there is limited data for reconstructing early hominin characteristics and their local paleoenvironments, the precise nature of these variations and selection pressures will likely remain unknown.

## 7. How Can We Test This Hypothesis

The tick-based hypothesis makes several predictions that can be tested experimentally. According to the hypothesis, hair loss coincided with paleoforest fragmentation, the most likely environmental context for hominin exposure to ticks. An early date for hominin hair loss (5–8 Ma) would support the tick-based hypothesis, whereas a recent date for hominin hair loss (~2 Ma) would essentially exclude the hypothesis. More precise data on the timing of hominin hair loss may be obtained by identifying the molecular mechanisms underlying hair loss and by determining the evolutionary timing of key genetic events [28]. For example, the hairless (*HR*) gene (chromosome 8p12) encodes a protein that has been implicated in certain types of congenital hair loss and has been found to play an important role in regulating the transition from the resting phase (telogen) to the growth phase (anagen) in postnatal hair follicle cycling. Recent studies show that the human *HR* gene underwent an increased rate of mutation following the human–chimpanzee divergence, with changes to eleven amino acids having potential structural or functional significance [320]. Further investigations comparing *HR* and other hair follicle regulatory genes (e.g., fibroblast growth factor 5 [*FGF5*]) and evidence of positive or relaxed selection pressures could help determine the evolutionary timing of hominin hair loss more precisely [321]. Similarly, investigations of brown fat physiology and its molecular and evolutionary basis in humans (e.g., via changes in uncoupling protein 1 [*UCP1*]) [322] could support the hypothesis that hair loss occurred in cool forest environments despite thermoregulatory disadvantages. Whole-genome interspecific comparative analyses of humans and chimpanzees could be used to identify genes or groups of genes under positive selection by the proposed exposure to ticks [323], with the prediction that genes related to hairlessness or grooming behaviour would show evidence of positive selection. Finally, it would be interesting to investigate whether LCA exposure to tick-borne disease 5–8 Ma may have resulted in a “catastrophic-selection” phenomenon similar to the abrupt loss of the α-gal epitope in ancestral apes and Old World monkeys, following the hypothesized exposure to an infectious agent 20–30 Ma [324,325,326].

The proposed relationship between chimpanzee grooming and ticks can be tested by observing anti-tick behaviours in wild chimpanzees [212] or by studying chimpanzee reward responses to ticks [202,244]. While endogenous opioids (e.g., β-endorphin) have been shown to play an important role in the motivation of primates to solicit social grooming [230], investigation into the motivation to perform social grooming could reveal alternative neurochemical pathways [e.g., possibly dopamine, given its role in reward-based mechanisms [327]]. The role of visualization in chimpanzee social grooming can be tested by observing the relationship between grooming behaviour and light [238], with the expectation that chimpanzees orient themselves to maximize their light source and that they avoid grooming in shadows. The study of terrestrial primates such as gorillas and François’ langurs, which have low social grooming times despite living on the ground, could reveal alternative non-grooming anti-tick strategies [328,329]. In addition, investigations into preening behaviour in humans (e.g., visually inspecting clothing for irregularities such as lint, leaves or dirt) [330] could support the tick-based hypothesis in which skin exposure due to hair loss may have allowed hominins to visually inspect themselves and others for ticks [215].

Finally, it is worth considering the possibility that the evolutionary split between hominins and chimpanzees may have resulted from a tick or tick-borne disease that disproportionately affected young LCAs (e.g., infants and juveniles). For example, Lyme disease in the United States shows a bimodal age distribution, disproportionately affecting young children (ages 5–9 years) and older adults (ages 50–55 years) [331]. Infant and juvenile chimpanzees share close social bonds with their mothers, who also function as their primary social groomers [31,197]. Therefore, an ancestral tick or tick-borne disease that disproportionately affected young LCAs could have selected for hairy children groomed by fastidious mothers (i.e., chimpanzees) or, alternatively, children that received low social grooming but had decreased body hair (i.e., humans). While genes for hair loss would affect the primary individual, genes for increased social grooming would more likely function altruistically for the benefit of the next generation [332]. These suggestions, albeit speculative, could be supported by considering the feeding patterns of ticks with regard to young primate hosts [213], the epidemiology of tick-borne diseases in children [333], and patterns of social grooming between primate mothers and their offspring [334].

## 8. Conclusions

The tick-based hypothesis presented here draws a strong connection with the mosaic environment associated with human evolution and explains why evolutionary changes observed in hominins did not occur in chimpanzees. The proposed processes are based on the logic of natural selection and are consistent with the principle of host diversification due to exposure to parasitic disease. The ecological relationships employed by the hypothesis are based on similar relationships observed today (i.e., taxonomic and ecological “uniformitarianism”). The hypothesis incorporates accepted data on tick ecology, hominin paleoenvironments, hominin hair loss, primate locomotion, and primate grooming habits. Finally, the tick-based hypothesis provides a basis for continued research into human origins and supports the view that evolutionary studies can benefit from considering a broad range of ecological, environmental, and social factors.

## Data Availability

There is no additional data for this manuscript.

## Supplementary Materials

The following are available online at https://www.mdpi.com/article/10.3390/life11050435/s1, Supplementary File S1. Minimal night-time temperatures at multiple sites surrounding Lake Victoria (weatherspark.com (accessed on 1 May 2021)). Supplementary File S2. Grooming time (% daily activity) in arboreal versus terrestrial primates.
