# Supplementary material for: Ticks, Hair Loss, and Non-Clinging Babies: A Novel Tick-Based Hypothesis for the Evolutionary Divergence of Humans and Chimpanzees
Source: Life (Basel). 2021 May 12;11(5):435. doi: 10.3390/life11050435 (PMC8150933; doi:10.3390/life11050435)
Supplement: Supplementary file 1 [file life-11-00435-s001.zip › Supplementary files 1 and 2_submitted.pdf]

Ticks, Hair Loss, and Non-Clinging Babies: A Novel Tick-Based Hypothesis for the Evolutionary Divergence of Humans and Chimpanzees

Jeffrey G. Brown

Supplementary file 1. Minimal night-time temperatures at multiple sites surrounding Lake Victoria (weatherspark.com)

Site	Country	Elevation (m)	Minimum Night-time Temperature Average/Range (°C)												Annual Lowest Night-time Temp (°C)
			Jan	Feb	Mar	April	May	Jun	Jul	Aug	Sept	Oct	Nov	Dec	
Mbarara	**Uganda**	**1420**	**15 (13–16)**	**15 (14–17)**	**15 (14–17)**	**15 (14–17)**	**15 (14–17)**	**15 (13–17)**	**15 (13–16)**	**15 (13–16)**	**15 (13–16)**	**15 (13–16)**	**15 (14–16)**	**15 (14–16)**	**13**
Kampala	**Uganda**	**1192**	**18 (16–19)**	**18 (16–20)**	**18 (17–20)**	**19 (17–20)**	**18 (17–20)**	**18 (16–19)**	**17 (16–18)**	**17 (16–18)**	**17 (16–18)**	**17 (16–19)**	**18 (16–19)**	**18 (16–19)**	**16**
Mbale	**Uganda**	**1130**	**18 (16–19)**	**18 (16–20)**	**18 (17–20)**	**18 (17–20)**	**18 (16–19)**	**17 (15–19)**	**17 (15–18)**	**17 (15–18)**	**17 (15–19)**	**17 (16–19)**	**17 (15–19)**	**17 (15–19)**	**15**
Bungoma	**Kenya**	**1422**	**15 (13–17)**	**15 (14–17)**	**16 (14–18)**	**16 (15–17)**	**16 (14–17)**	**15 (13–16)**	**15 (13–16)**	**15 (13–16)**	**15 (13–16)**	**15 (14–17)**	**15 (14–17)**	**15 (14–17)**	**13**
Kakamega	**Kenya**	**1526**	**15 (13–17)**	**15 (13–17)**	**16 (14–17)**	**16 (14–17)**	**15 (14–17)**	**15 (13–16)**	**14 (13–16)**	**14 (13–16)**	**14 (13–16)**	**15 (13–16)**	**15 (14–16)**	**15 (13–17)**	**13**
Migori	**Kenya**	**1438**	**16 (14–17)**	**16 (14–17)**	**16 (14–18)**	**16 (14–17)**	**15 (14–17)**	**15 (13–16)**	**15 (13–16)**	**15 (13–16)**	**15 (14–17)**	**16 (14–17)**	**16 (14–17)**	**16 (14–17)**	**13**
Musoma	**Tanzania**	**1150**	**20 (18–21)**	**20 (18–21)**	**20 (19–22)**	**20 (18–21)**	**19 (18–21)**	**19 (17–20)**	**18 (16–20)**	**19 (17–20)**	**19 (18–21)**	**19 (18–21)**	**19 (18–21)**	**19 (18–21)**	**16**
Mwanza	**Tanzania**	**1181**	**19 (17–20)**	**19 (17–21)**	**19 (18–21)**	**19 (18–21)**	**18 (16–20)**	**17 (15–19)**	**17 (15–19)**	**18 (15–20)**	**19 (16–20)**	**19 (18–21)**	**19 (18–21)**	**19 (17–20)**	**15**
Shinyanga	**Tanzania**	**1132**	**16 (14–18)**	**16 (14–18)**	**17 (15–19)**	**17 (15–19)**	**16 (14–19)**	**15 (13–18)**	**15 (12–17)**	**16 (13–18)**	**17 (14–19)**	**18 (15–20)**	**18 (16–20)**	**17 (15–19)**	**12**
Ushirombo	**Tanzania**	**1197**	**16 (14–18)**	**16 (15–18)**	**17 (15–19)**	**17 (16–19)**	**17 (14–19)**	**15 (12–18)**	**15 (12–17)**	**16 (13–18)**	**17 (14–19)**	**17 (15–19)**	**17 (15–19)**	**16 (15–18)**	**12**
Bukoba	**Tanzania**	**1197**	**18 (16–20)**	**18 (17–20)**	**19 (17–21)**	**19 (17–21)**	**19 (17–20)**	**18 (16–20)**	**17 (16–19)**	**18 (16–19)**	**18 (16–19)**	**18 (17–19)**	**18 (17–20)**	**18 (17–20)**	**16**
Nsunga	**Tanzania**	**1202**	**16 (14–17)**	**16 (14–18)**	**16 (15–18)**	**17 (15–18)**	**16 (14–18)**	**16 (14–18)**	**15 (14–17)**	**16 (14–17)**	**16 (14–17)**	**16 (14–17)**	**16 (15–18)**	**16 (14–17)**	**14**

Supplementary file 2. Grooming time (% daily activity) in arboreal versus terrestrial primates

Species	Common Name	Grooming Time (% Daily Activity)	Average Grooming Time (% Daily Activity)
**Arboreal**			
	**Orangutans†**		
Pongo pygmaeus	Bornean orangutan	0.01[1]	**Orangutans**
Pongo abelii	Sumatran orangutan	0.01[1]	
		**0.02/2 =**	**0.01**
	**New World Monkeys**		
Saguinus mystax	Moustached tamarin	1.85[1]	
Cebus olivaceus	Wedge-capped capuchin	1.9[1]	
Cebus apella	Tufted capuchin	1.21[1]	
Cebus albifrons	White-fronted capuchin	0.615[1]	
Callicebus torquatus	Collared titi	9.9[1]	
Callice oenanthe	Rio Mayo titi	4.5[1]	
Chiropotes satanas	Black bearded saki	0.4[1]	
Pithecia pithecia	White-faced saki	0.8[1]	
Cacajao calvus	Bald uakari	1.8[1]	
Alouatta palliata	Mantled howler monkey	0[1]	
Alouatta seniculus	Venezuelan red howler	0.4[1]	
Alouatta guariba	Brown howler	2.6[1]	
Alouatta caraya	Black howler	1.2[1]	
Ateles geoffroyi	Black-handed spider monkey	2.5[1]	
Ateles belzebuth	White-bellied spider monkey	0.1[1]	
Brachyteles hypoxanthus	Northern miriqui	0[1]	**New World Monkeys**
		**29.8/16 =**	**1.9**
	**Gibbons/Siamangs**		
Hylobates lar	Lar gibbon	4.4[1]	

Hylobates klossii	Kloss's gibbon	0[1]	**Gibbons/ Siamangs**
Hylobates agilis	Agile gibbon	0[1]	
Hylobates pileatus	Pileated gibbon	5[1]	
Symphalangus syndactylus	Siamang	10[1]	
		**19.4/5 =**	**3.9**

Arboreal Old World Monkeys

Presbytis siamensis	White-thighed surili	0[1]
Presbytis potenziani	Mentawai langur	0.1[1]
Presbytis rubicunda	Maroon leaf monkey	0[1]
Presbytis thomasi	Thomas's langur	1.3[1]
Trachypithecus pileatus	Capped langur	0.4[1]
Rhinopithecus avunculus	Tonkin snub-nosed monkey	5.6[1]
Nasalis larvatus	Proboscis monkey	2.8[1]
Pygathrix nigripes	Black-shanked douc	2.25[1]
Pygathrix nemaeus	Red-shanked douc	1.78[1]
Colobus guereza	Mantled guereza	6.2[1]
Colobus satanas	Black colobus	5.5[1]
Colobus polykomos	King colobus	0.7[1]
Colobus angolensis	Angola colobus	5[1]
Colobus vellerosus	Ursine colobus	1[1]
Piliocolobus rufomitratus	Tana River red colobus monkey	2.1[1]
Piliocolobus badius	Western red colobus	5.4[1]
Piliocolobus kirkii	Zanzibar red colobus	7[1]
Piliocolobus tephrosceles	Ugandan red colobus	5.2[1]
Procolobus verus	Olive colobus	3.6[1]
Cercopithecus mitis	Blue monkey	8[1]
Cercopithecus ascanius	Red-tailed monkey	5.6[1]
Cercopithecus campbelli	Campbell's mona monkey	2.8[1]
Cercopithecus diana	Diana monkey	2.5[1]

Chlorocebus djamdjamensis	Bale monkey	2.7[1]	
Lophocebus albigena	Grey-cheeked mangabey	5.2[1]	
Cercocebus galeritus	Tana river mangabey	5.5[1]	
Macaca fascicularis	Crab-eating macaque	10.6[1]	**Arboreal Old World Monkeys**
		**98.8/27 =**	**3.7**

Arboreal Lemurs

Avahi laniger	Eastern wooly lemur	2[1]	
Eulemur fulvus	Common brown lemur	8.3[1]	
Propithecus verreauxi	Verreaux's sifaka	2[1]	**Arboreal Lemurs**
		**12.3/3 =**	**4.1**

Terrestrial

Chimpanzees†

Pan paniscus	Bonobo	5.7[1]	
Pan troglodytes	Common chimpanzee	9.8[1]	**Chimpanzees**
		**15.5/2 =**	**7.8**

Terrestrial Old World Monkeys

Trachypithecus leucocephalus	White-headed langur	11.5[1]
Trachypithecus francoisi	François' langur	1.2[1]
Semnopithecus achates	Southern plains gray langur	6[1]
Semnopithecus entellus	Gray langur	6[1]
Semnopithecus cf. schistaceus	Nepal gray langur	8.4[1]
Rhinopithecus bieti	Black snub-nosed monkey	6.7[1]
Chlorocebus aethiops	Vervet monkey	6.8[1]

Erythrocebus patas	Patas monkey	4.53[1]	
Macaca fuscata	Japanese macaque	18[1]	
Macaca mulatta	Rhesus macaque	6.6[1]	
Macaca munzala	Arunachal macaque	12[1]	
Theropithecus gelada	Gelada	17.4[1]	
Papio anubis	Olive baboon	7.1[1]	
Papio papio	Guinea baboon	8.3[1]	
Papio hamadryas	Hamadryas baboon	13.5[1]	**Terrestrial Old World Monkeys**
Papio ursinus	Chacma baboon	12.8[1]	
		**146.8/16 =**	**9.2**
**Terrestrial Lemurs**			**Terrestrial Lemurs**
Lemur Catta	Ring-tailed Lemur	**7.8[1]/1 =**	**7.8**
**Gorillas†**			
Gorilla gorilla	Western gorilla	0.1[1]	**Gorillas**
Gorilla beringei	Eastern gorilla	2.5[1]	
		**2.6/2 =**	**1.3**
**Humans†‡**			**Humans**
Homo sapiens	Human	**0.8[2]/1 =**	**0.8**

†Hominid. ‡Hominin.
1 Grueter, C.C., et al. (2013, Ref. 220).
2 Jaeggi, A.V., et al. (2017, Ref. 221).
